# Supplementary material for: Are carbon emissions trading and green financial instruments synergistic? -Comprehensive quantitative research based on content analysis
Source: PLoS One. 2024 Mar 7;19(3):e0298601. doi: 10.1371/journal.pone.0298601 (PMC10919601; doi:10.1371/journal.pone.0298601)
Supplement: S1 Data — (ZIP) [file pone.0298601.s001.zip › Data/地级市Z_F工作报告.zip!/║■▒▒/╧σ╤⌠╩╨/└┤╫╘╣1⁄2╓┌║┼í╛┬φ┐╦╩2╛▌═°í┐_2016.docx]

各位代表：

　　现在，我代表市人民政府向大会报告工作，请与《襄阳市国民经济和社会发展第十三个五年规划纲要（草案）》一并审议，并请市政协委员和其他列席人员提出意见。

　　一、“十二五”时期经济社会发展回顾

　　“十二五”时期是我市发展进程中极不平凡的五年。全市上下在市委的坚强领导下，主动适应新常态，克难攻坚促跨越，“两个中心、四个襄阳”建设持续突破，“十二五”规划确定的主要目标任务全面完成，襄阳面貌发生了显著变化。

　　——这五年，经济发展逆势跨越，综合实力显著提升。地区生产总值跃上3000亿元台阶，预计2015年突破3400亿元，是2010年的2.2倍，占全省的比重由“十一五”期末的9.7%提高到11.4%；地方公共财政预算收入、规模以上工业总产值跃居全省第二位，分别达到339.1亿元和6000亿元，分别是2010年的6.6倍和2.6倍。

　　——这五年，城市能级蝶变跃升，竞争优势显著扩大。城市格局发生了重大变化，“一心四城”的空间形态基本形成；城市品质明显提升，“一城两文化”特色日益彰显，荣膺国家森林城市、国家卫生城市；区域性综合交通枢纽加速形成，区域性铁路枢纽、国家公路运输枢纽的地位不断强化，航空港吞吐能力显著增强；城市影响力明显扩大，地区生产总值和地方公共财政预算收入跃居汉江流域城市第一位，跻身中国社会科学院“城市竞争力百强”排行榜第74位。

　　——这五年，改革红利加速释放，发展动能显著增强。80多项全国全省改革创新试点示范在我市实施，制约经济社会发展的一些体制机制障碍得到有效破解，大众创业万众创新动能加速集聚，市场主体迅猛增长，五类市场主体发展到42.8万户，比2010年净增27.5万户。

　　——这五年，发展方式转变加快，发展质量显著提高。区域发展日趋协调，7个县（市、区）在全省县域经济考核中全线进位，襄阳高新区工业总产值在147个国家级高新区中跃居第20位；创新驱动力不断增强，我市成为全国科技进步示范市、国家创新型试点城市、国家可持续发展实验区、国家现代农业示范区；生态环境质量持续改善，汉江干流襄阳段水质稳定在Ⅱ类水平。

　　——这五年，民生福祉不断增进，人民生活显著改善。基本民生保障水平持续提升，养老、医疗、救助等社会保障体系实现全覆盖，25.5万贫困人口稳定脱贫，连续四年在全省扶贫考核中获得优秀，160多万农村人口饮水安全问题得到解决，88万城乡居民通过保障性住房建设和农村危房改造实现“居有其屋”。人民生活不断改善，城乡居民人均可支配收入实现倍增；人民群众安全感逐年提升，我市成为全国社会管理综合治理优秀市。

　　刚刚过去的2015年，我市经济社会发展取得了显著成绩，预计地区生产总值比上年增长9%，规模以上工业增加值增长9.8%，全社会固定资产投资增长20%，社会消费品零售总额增长12.6%；地方公共财政预算收入增长36.1%，城镇和农村常住居民人均可支配收入分别增长7%和8%，计划生育、节能减排、安全生产、居民消费价格指数等约束性指标全面完成省定目标。

　　五年来，我们主要做了以下五个方面的工作：

　　(一)以扩大有效投资强化跨越赶超支撑。预计五年累计完成全社会固定资产投资过万亿，是“十一五”时期的4.6倍。强化重大项目的支撑作用，累计实施省市重点项目785个，完成投资3842亿元，其中投资10亿元以上的项目224个。强化招商引资的带动作用，累计完成招商项目投资5344亿元，新引进世界500强企业22家。强化工业投资的主导作用，累计完成投资5276亿元，是“十一五”时期的4.4倍，鸿泰阳新能源汽车、中车工业园等一批重大工业项目相继开工，神龙公司扩能、骆驼集团二期等755个亿元以上项目建成投产。强化支撑性工程的基础作用，累计完成交通投资471亿元，是“十一五”时期的6.8倍，汉十高铁和蒙华铁路开工建设，襄阳机场改扩建、襄阳新港建设快速推进，实现了县县通高速、镇镇通二级以上公路、村村通水泥路；累计完成水利工程投资68.4亿元，是“十一五”时期的2.2倍，鄂北水资源配置、水库除险加固、大中型灌区续建配套等一批重大水利工程相继实施。

　　（二）以产业转型升级提升发展质量效益。着力推进万亿工业强市建设，新增农产品加工、装备制造2个千亿级产业，培育全省重点成长型产业集群10个，新增中国驰名商标39个、中国地理标志商标27个，战略性新兴产业总产值占规模以上工业的比重提高到六分之一，产销过亿元的企业达到1100家，其中6家过100亿、97家过10亿，风神公司成为襄阳首家产值突破300亿元的企业，骆驼股份、襄大农牧跻身“中国民营企业500强”，我市成为国家新型工业化（新能源汽车、军民结合、再生资源利用）产业示范基地。着力推进现代服务业跨越发展，云计算、电子商务、现代物流等新兴服务业快速壮大，“襄阳云谷”成为全省信息化支撑平台，我市成为国家二级物流园区布局城市、全国城市共同配送试点城市、国家电子商务示范城市创建试点；旅游“二次创业”取得重大进展，“千古帝乡、智慧襄阳”品牌影响力日益扩大。着力推进现代农业强市建设。农业综合生产能力显著提升，新型农业经营主体快速成长，“中国有机谷”建设取得积极进展，我市进入全国百亿斤粮食生产大市行列，连续四年获得“全国粮食生产先进市”称号。

　　（三）以新型城镇化引领城乡一体发展。着力优化新型城镇化发展格局，“四位一体”的市域城镇体系初步建立，常住人口城镇化率达到57.1%，比2010年提高7.1个百分点。着力推进新区建设，东津新区核心区基础设施实现全覆盖，起步区骨干路网基本形成，公共工程建设有序推进；各县（市）新区核心区基础设施网络基本建成。着力推进旧城更新，改造棚户区16.63万户，综合整治“三无”小区510个、惠及20万市民。着力推动城市功能升级，完成了长虹路、襄樊大道改造，建成了卧龙大桥、东津大桥和胜利街立交桥，内环线全线贯通，城市快速路网体系初步建立，主次干道通行能力明显提升，公交线网覆盖面不断扩大，燃气普及率、公共供水普及率、生活污水集中处理率大幅提高，雨污分流管网覆盖面扩展到80平方公里。着力打造山水园林名城，深入实施文化融入、“九水润城”、城市亮化和生态绿地建设工程，新建改建公园游园20个，新增绿地400万平方米、绿道120公里。着力推进城管革命，建立健全“大城管”体制机制，大力开展“五城同创”，城市精细化管理水平不断提高。着力推进城乡一体化，宜城全国新型城镇化综合改革试点扎实开展，“四化同步”示范镇和美丽乡村建设成效显著，103个村镇成为全国、全省试点示范。着力加强生态文明建设，“蓝天碧水”、“绿满襄阳”工程深入实施，节能减排目标任务全面完成，城市污泥综合处置示范项目获得“中国人居环境范例奖”。

　　（四）以改革创新激发经济社会发展动力活力。深入推进重点领域和关键环节改革。行政管理体制改革取得重大突破，在全省率先建立“一局两委”、“三个一”、“三张清单”、“三零服务”为主的行政管理新体制，率先实施“先照后证”、“三证合一、一照一码”为核心的商事制度改革，有力促进了简政放权；投融资体制改革深入推进，各类金融机构和业态加速集聚，在全省率先设立产业基金和城市发展基金，金融机构、上市公司和新三板、四板挂牌企业数量均居全省市州前列，打造了全省四板市场首个市域科技板互联网板块；农村改革扎实推进，农村土地确权登记颁证有序实施，市、县农村产权交易体系全面建立，集体林业综合改革、农村公共服务维护机制建设等工作成为全国示范；土地管理制度创新、户籍制度改革、综合执法体制改革、医药卫生体制改革、文化体制改革等都取得了显著成效。着力构建开放型经济体系。湖北自贸区襄阳片区筹建工作取得积极进展，“三港一中心”建设取得实质性突破，襄阳保税物流中心获得国家批准；外向型经济发展加快，出口额由全省第6位跃升至第3位，新批外商投资企业125家。着力提升科技创新能力。新增国家级和省级研发创新平台112个，获得省级以上重大科技成果1219项、授权专利8983件，高新技术企业新增217家、达到290家；建成国家和省级各类孵化器9个、省级众创空间7个；“隆中人才支持计划”深入实施，引进海内外高层次创新创业团队25个。

　　（五）以加强社会建设提高民生保障水平。持续加大民生投入，全市民生支出占一般公共预算支出的比重达到74.3%；大力实施改善民生三年行动计划，解决了一批人民群众最关心最直接最现实的利益问题。着力提高就业和社会保障水平，新增城镇就业52万人，基本养老和医疗保险覆盖面不断扩大，大病医疗保险覆盖所有参保人口，城乡低保和农村“五保”对象实现应保尽保、保障待遇大幅提高。着力提高教育发展水平，学前教育公共服务体系基本建成，各县（市、区）均通过了省级义务教育均衡发展评估验收，普通高中教育质量全省领先，襄阳四中、襄阳五中跻身“全国百强高中”行列，湖北文理学院创建综合性大学工作扎实推进，襄阳职业技术学院成为“中国职业教育百强院校”。着力完善公共文体服务体系，四级公共文化体育设施网络基本建成，文化惠民工程深入推进，“欢乐襄阳”等群众文化品牌影响力不断扩大。着力增强卫生计生服务能力，创建国家级和省级临床重点专科、重点学科25个，建成省级“四化”乡镇卫生院51家、“五化”村卫生室1682个，公共卫生服务保障能力明显增强，重大传染病得到有效防控；坚持计划生育基本国策，省定计划生育指标全面完成。着力提升社会治理水平，“六五”普法深入实施，城乡网格化管理实现全覆盖，安全生产管理、食品药品安全监管、流通领域商品质量管理、立体化社会治安防控体系不断完善，法治湖北现场会在我市召开，法治襄阳建设经验在全省推广，我市成为国家安全发展示范试点城市、国家食品安全试点城市。国防动员、“双拥”共建、民族宗教、对口支援等工作都取得了显著成绩。

　　五年来，我们不断加强政府自身建设，深入推进依法行政，法治政府建设走在全省前列；积极推进政务公开，建立权力清单制度，促进了行政权力公开透明运行；强化行政执法监督，加强行政复议和行政诉讼应诉工作，促进了规范公正文明执法；坚持重大事项向人大报告、与政协协商制度，自觉接受市人大及其常委会的法律监督、工作监督和市政协的民主监督；扎实开展党的群众路线教育实践活动和“三严三实”专题教育，深入开展“假如我是服务对象”大讨论活动，推进市民问政、治庸问责常态化，有力促进了行政机关作风转变、效能提升；加强党风廉政建设，严格落实中央八项规定精神，弛而不息整治“四风”，大幅压缩“三公”经费支出，强化政府投资项目和财政资金监管，规范公共资源配置交易，促进了廉洁政府建设。

　　过去五年取得的成就，为率先全面建成小康社会奠定了坚实基础，极大地增强了全市人民的自信心和自豪感，必将激励我们在新的征程中奋勇前进。这些成就，是市委带领全市广大干部群众认真贯彻省委、省政府决策部署，顽强拼搏、共同奋斗的结果。在此，我代表市政府，向全市人民，向各位人大代表、政协委员，向各民主党派、工商联、人民团体和无党派人士，向驻襄人民解放军、武警官兵和公安干警，向所有关心支持襄阳发展的同志们、朋友们，表示衷心的感谢，致以崇高的敬意！

　　回顾“十二五”时期的工作，我们深切体会到，推动襄阳发展、造福襄阳百姓，必须坚持人民至上，把人民群众对美好生活的向往作为奋斗目标，把增进人民群众福祉作为发展的出发点和落脚点；必须坚持奋发竞进，在湖北“建成支点、走在前列”大局中勇挑重担，在区域发展竞争中抢前争先；必须坚持解放思想，勇于打破惯性思维、突破传统模式，以改革创新的举措集聚跨越赶超新动能；必须坚持高点定位，在全国全省发展大局中定坐标，以科学规划引领发展；必须坚持善作善成，紧紧盯住打基础、利长远的大事要事，重锤钉钉子、蚂蚁啃骨头，持之以恒、久久为功。

　　二、“十三五”时期的形势和任务

　　根据《中共襄阳市委关于制定全市国民经济和社会发展第十三个五年规划的建议》，我们编制了《襄阳市国民经济和社会发展第十三个五年规划纲要（草案）》，提交大会审议。

　　“十三五”时期，是我市率先全面建成小康社会的决胜阶段，是实现产业层级、经济量级、城市能级和社会治理水平快速跃升的关键时期。科学谋划和推进“十三五”发展，必须冷静分析形势，准确研判走势。

　　面向“十三五”发展，我们必须清醒认识面临的压力和挑战。发展不够仍然是襄阳最大的实际，实现经济总量占全省六分之一的目标差距很大，壮大经济实力的任务紧迫而艰巨；传统产业比重偏大，战略性新兴产业规模偏小，现代服务业支撑不足，农产品加工业层次不高，推进产业升级的任务紧迫而艰巨；创新能力不强、创业活力不足、开放开发水平不高等问题还比较突出，发展动力转换的任务紧迫而艰巨；区域中心城市的支撑性功能不足，县级城市和中心城镇承载功能不强，推进城乡一体协调发展的任务紧迫而艰巨；资源环境约束趋紧，尤其是保护和改善水环境、大气环境、农村环境，推进绿色发展的任务紧迫而艰巨；基本公共服务和社会保障能力与人民群众的期待还有很大差距，贫困人口全面脱贫的压力很大，率先全面建成小康社会的任务紧迫而艰巨。

　　面向“十三五”发展，我们必须牢牢把握各种机遇和有利条件。从战略层面看，襄阳纳入了长江经济带建设、长江中游城市群、大别山革命老区振兴发展等国家重大战略规划，汉江生态经济带建设上升为国家战略，国家将在中西部地区培育一批区域性中心城市，省委、省政府强力实施“一主两副多极”发展战略，这些战略性机遇交汇叠加，必将把襄阳带入一个黄金发展期；从重大项目布局看，郑万高铁、汉十高铁、蒙华铁路、汉江航运、鄂北水资源配置工程、大唐热电联产、华电三期、路口电站、棚户区改造、汉江生态建设等一大批重大投资项目列入了国家规划，将在“十三五”时期全面实施、陆续建成，不仅会强力拉动经济增长，而且将推动襄阳的基础设施、城市能级实现质的跃升；从发展基础看，近几年相继开工建设和竣工投产了一批重大产业项目，为“十三五”的发展积蓄了强大能量、奠定了坚实基础。

　　面向“十三五”发展，我们必须科学把握和主动适应经济发展新常态。面对增长速度换档的趋势，必须坚持增量与提质并举，在提高发展质量和效益的基础上能搞多快就搞多快，努力做到竞进提质、又好又快；面对发展方式转变的趋势，必须坚持加快发展与保护环境并重，促进形成绿色生产方式和生活方式，努力做到经济增长与资源环境相协调；面对动力机制转换的趋势，必须坚持改造提升传统产业与培育壮大新兴产业相结合，加快推进供给侧结构性改革，努力提高投资的有效性，激发创新创业活力，促进新技术、新产业、新业态蓬勃发展。

　　综合分析“十三五”发展面临的形势，困难挑战很大，机遇千载难逢，我们务必增强忧患意识、责任意识、使命意识，牢牢把握新常态下抓发展的主动权，着力在优结构、强动力、补短板、壮实力上下功夫，不断开拓发展新境界。

　　“十三五”时期全市经济社会发展的指导思想是：全面贯彻党的十八大和十八届三中、四中、五中全会精神，以习近平总书记系列重要讲话精神为统领，按照“四个全面”战略布局的要求，牢固树立“五大发展理念”，落实省委十届七次全会部署和“三维纲要”精神，围绕建设汉江流域中心城市、率先全面建成小康社会的奋斗目标，更加注重经济转型升级和社会转型发展，更加注重创新创业和改革开放，更加注重依法治理和文化引领，更加注重城乡一体和区域合作，更加注重生态文明建设和持续改善民生，确保县域经济在全省考核中争先进位，确保经济增速持续领跑全省和汉江流域，确保主要经济指标占全省及汉江流域的比重持续提升，确保城乡居民收入与经济同步增长、共享改革发展成果，为湖北“建成支点、走在前列”作出更大贡献。

　　“十三五”时期全市经济社会发展的总体目标是：

　　——经济发达，综合实力大幅提升。全力冲刺经济总量占全省六分之一的目标，到2020年，全市地区生产总值力争突破6000亿元，规模以上工业总产值突破1.2万亿元，地方公共财政预算收入突破600亿元，成为汉江生态经济带开放开发的战略引擎。

　　——文化繁荣，名城效应全面彰显。建成国家公共文化服务体系建设示范区，文化创造力、文化影响力显著提升，“一城两文化”特色充分彰显，成为全国文明城市。

　　——法治优良，城乡社会和谐有序。社会治理体系和治理能力现代化水平显著提升，法治政府建设不断加强，社会公平正义得到切实维护，城乡社会既充满活力又和谐有序，成为“全省领先、全国一流”的法治城市。

　　——功能完善，城镇建设提档升级。新型城镇化和城乡发展一体化走在汉江流域城市前列，全市常住人口和户籍人口城镇化率分别达到62%、55%。中心城区建成区面积达到200平方公里、人口达到200万人，形成布局合理、配置高效、承载有力的功能体系。山水园林城市特色充分彰显，成为全国生态园林城市。

　　——生态一流，绿色发展创造示范。生态环境质量持续改善，生产方式和生活方式绿色、低碳水平明显上升，成为全国可持续发展先进示范区、生态保护和建设示范区。

　　——人民幸福，群众生活更加殷实。城乡居民收入率先翻一番，在全省率先实现脱贫目标，实现劳有厚得、学有优教、病有良医、老有颐养、住有宜居，人民群众生活水平和质量明显提高，获得感、幸福感显著增强。

　　围绕实现上述目标，“十三五”时期将突出抓好以下六项重点任务：

　　（一）构建现代产业新体系

　　建设万亿工业强市。坚持放手发展规模，努力提高质量，实施中国制造2025襄阳行动计划，全面落实《关于加快推进万亿工业强市建设的意见》，推动工业量级和产业层级“双跃升”，工业经济总量三年突破万亿、五年实现倍增，加快建设具有比较竞争优势的先进制造业基地，着力打造“中国新能源汽车之都”、国家军民融合创新示范区。到2020年，汽车及零部件产业突破4000亿元，农产品加工业突破3000亿元，装备制造产业突破2000亿元，电子信息、医药化工、纺织服装产业分别突破1000亿元，节能环保、新能源新材料产业分别突破600亿元，军民融合产业板块产值突破1000亿元。高新区工业总产值突破5500亿元，枣阳市、襄州区分别突破1500亿元，经开区、宜城市、谷城县、老河口市分别突破1000亿元。新认定高新技术企业300家以上，战略性新兴产业增加值和高新技术产业增加值占GDP的比重分别达到15%和26%左右。

　　建设现代农业强市。以整市推进国家现代农业示范区和“中国有机谷”建设为抓手，加快转变农业发展方式，推动一二三产业融合发展，着力构建现代农业生产体系、产业体系、经营体系，全面提高农业质量效益和竞争力。

　　建设现代服务业强市。推动生产性服务业向专业化和价值链高端延伸，生活性服务业向精细化和高品质转变，促进现代服务业与先进制造业、现代农业融合发展，加快壮大现代服务业规模，着力打造区域性现代服务业中心。

　　（二）建设先进基础设施

　　提升综合交通枢纽功能。提升铁路枢纽功能，全力支持和服务蒙华、汉十、郑万三大铁路建设，高标准规划建设东津高铁枢纽。完善国家公路运输枢纽功能，实现所有县（市）通2条以上高速公路，中心城区至各县（市）实现一级以上公路快速联通，干线公路与县乡等级公路连结成网，县乡等级公路与通村沥青水泥路条条相通，3A级以上景区通二级以上公路。加快推进汉江梯级开发，打造汉江航运中心。建设区域性航空服务中心，把襄阳机场建成一类航空口岸和区域性门户机场。

　　提升能源保障能力。实施一批清洁能源和可再生能源开发项目，加强城市电网配套建设和农村电网升级改造，加快建设新能源汽车充电设施网络。完善中心城区天燃气管网及配套设施，推进燃气管网向县（市）城区覆盖、向重点中心镇延伸。

　　提升信息网络建设水平。推进宽带网络优化升级，提升“光网城市”、“无线城市”、“智慧城市”、“三网融合”建设水平，构建功能强大的公共信息网络体系。

　　提升水利基础设施建设水平。加快推进鄂北水资源配置工程及配套工程建设，确保同步建成、同步受益。持续推进大中型灌区续建配套与节水改造，提高防汛抗旱减灾能力。加强城乡供水设施及城市备用水源建设，提高饮水安全保障能力。

　　（三）构建城乡一体新型城镇化发展格局

　　优化市域城镇化发展布局。坚持以新型城镇化引领城乡一体化发展，完善市域城镇体系。科学划定城市发展边界，优化提升中心城区“一心四城”空间形态和功能布局，以东津新区为核心积极创建国家级汉江新区。构建区域协调发展新格局，壮大枣阳副中心支撑作用，加快推进襄宜南、河谷一体化发展，支持保康生态旅游试验区建设。

　　全面提升中心城市辐射带动功能。加快推进高新区产城融合发展，强化对全市经济的核心引擎作用。强力推进东津新区建设，基本建成70平方公里起步区。有序推进庞公新区、樊西新区建设，实现核心区基础设施全覆盖。大规模推进棚户区改造，五年内全面完成中心城区棚户区改造任务。按照绿色低碳循环理念规划建设城市基础设施，完善城市交通体系，规划并力争开工建设城市轨道交通；完善供水、排水、治污等市政设施，实施“海绵城市”建设，加快建设城市综合管廊系统。

　　大力推进城市绿色发展。着力建设绿色城镇，把好山好水好风光融入城市，大幅拓展绿色空间，大力推广绿色交通、绿色建筑、绿色设施，打造山清水秀的美丽家园；着力建设人文城镇，坚持以强烈的文化意识引领城镇化和城乡一体化发展，加大古城、历史文化街区、风景名胜区和传统村落保护力度，打造一批有历史记忆、文化脉络、地域风貌的特色城镇和村庄；着力建设智慧城镇，加快建设智慧化城市管理运行体系。

　　健全完善城镇化和城乡一体化发展体制机制。统筹推进城乡发展规划、产业布局、资源配置、基础设施、公共服务、就业社保和社会管理等“七个一体化”，推动城镇公共服务向农村延伸。深化户籍制度改革，实现城镇基本公共服务常住人口全覆盖。

　　（四）建设宜居宜业美丽襄阳

　　优化国土空间开发格局。落实国家主体功能区规划，统筹各类空间规划，推进“多规合一”。按照生产空间集约高效、生活空间宜居适度、生态空间山清水秀的总体要求，构建科学合理的城市化格局、农业发展格局、生态安全格局。

　　推动绿色低碳循环发展。大力推广清洁能源，实施燃煤锅炉燃气替代工程，提高非化石能源利用比重。实施循环发展引领计划，全面推进工业园区循环化改造、传统制造业绿色化改造，大力推行农业循环发展模式，加快建立城市废弃物无害化处理资源化利用体系，打造再生资源利用产业链，支持谷城国家循环经济示范县建设。实施全民节能行动计划，落实最严格的耕地保护和水资源管理制度。

　　加大环境治理力度。实施大气、水、土壤污染防治行动计划，实现工业污染源全面达标排放，确保城镇生活污水处理设施全覆盖和稳定运行。加大农业面源污染防治力度，统筹实施农村饮水安全、改水改厕、垃圾处理等工程，持续改善农村人居环境，推进美丽乡村建设。

　　筑牢生态安全屏障。着力构建市域“一核三带九廊多点”的森林生态系统，加快建设中心城区“一心六片九水”的生态体系。深入推进全国水生态文明试点城市建设，加大水环境保护力度，确保汉江干流襄阳段水质稳定在Ⅱ类水平。

　　（五）培育创新发展新优势

　　深入实施创新驱动发展战略。以打造区域性科技创新中心为目标，大力支持企业开展自主创新、协同创新，建设一批高水平研发机构，培育一批具有核心竞争力的创新型企业，组建一批产业技术创新联盟。大力推进科技成果转化，加快建设襄阳科技城、院士专家创新创业园、华科工研院、海智基地等科技成果转化基地。实施人才优先发展战略，推进人才发展体制改革和政策创新，更大规模引进高层次人才和创新创业团队。

　　大力推进大众创业万众创新。着力构建有利于“双创”的体制机制、政策环境、众创空间，打造国内机制最活、生态最好、文化最优的创新之城、创业之城、创客之城，市场主体总量达到55万户以上。

　　持续释放改革红利。深化行政管理体制改革，持续推进简政放权、放管结合，构建权责明确、透明高效的监管机制。深入推进财税管理体制改革，建立全面规范、公开透明的现代预算制度，实行中期财政规划管理制度，深化税收征管体制改革。深入推进金融改革创新，加快建设多层次资本市场，培育具有襄阳特色的要素交易市场，打造汉江流域金融服务中心。深入推进投融资体制改革，积极推广运用政府与社会资本合作模式。深化土地管理制度改革，促进土地集约节约利用。深化国有企业改革，建立健全国有资产监管体系。深化农村集体产权制度改革，推进集体林业综合改革和国有林场改革，充分释放农业农村发展活力。深化公共服务提供方式改革，大力推广政府购买服务。深化生态文明体制改革，建立系统完整的生态文明制度体系。创新社会治理体制机制，深入推进法治襄阳建设，营造和谐稳定的社会环境。

　　建设汉江流域对外开放高地。加快构建对外开放大通道、大平台、大通关，打造“一带一路”通道上的重要节点城市，建设国际陆港开发区，融入全国通关一体化大格局。加快对外贸易优化升级，大力引进技术领先的项目、业态先进的产业，大力支持优秀企业到境外投资，深度融入全球产业链、物流链；实施商标“走出去”战略，引导品牌企业国际化发展。完善对外开放服务体系，打造法治化、国际化、便利化的营商环境。

　　（六）增进人民群众福祉

　　增加公共服务供给。提高教育质量，促进教育公平，着力打造区域性教育中心。完善城乡公共文化体育服务网络，推动基本公共文化服务标准化、均等化发展。加快建设汉江流域医疗卫生中心，深化医药卫生体制改革，健全公共卫生和基层医疗卫生服务体系，创建国家健康城市。促进人口均衡发展，坚持计划生育基本国策，加快建设多层次养老服务体系，保障妇女和未成年人权益，支持残疾人事业发展。

　　实施脱贫攻坚三年行动计划。按照“精准扶贫、不落一人”的总要求，坚决打赢脱贫攻坚战，确保到2017年底四个插花贫困市（区）建档立卡扶贫对象稳定脱贫、贫困村出列，到2018年底三个山区县建档立卡扶贫对象稳定脱贫、贫困村出列，实现“摘帽”目标。

　　大力促进就业创业。强化政府就业公共服务责任，实施更加积极的就业政策，完善就业创业服务体系，加强就业创业培训，努力为人民群众创造更多更稳定的就业岗位。

　　加快建立可持续的社会保障制度。实施全民参保计划，实现人人享有社会保障。健全社会救助体系，确保困难群众基本生活。建立健全多层次住房保障体系。

　　三、2016年重点工作

　　2016年是“十三五”的起步之年，我们将认真贯彻中央和省经济工作会议、城市工作会议精神，扎实做好今年的各项工作，奋力实现“十三五”发展的良好开局。

　　今年全市经济社会发展的主要预期目标是：地区生产总值增长10%，规模以上工业增加值增长10%以上，全社会固定资产投资增长20%，社会消费品零售总额增长13.5%以上，地方公共财政预算收入增长13%，实际外商直接投资增长15%，出口总额增长15%，城镇和农村常住居民人均可支配收入分别增长10%，全面完成精准脱贫、计划生育、安全生产、节能减排、居民消费价格指数等省定目标。

　　围绕实现预期目标，重点做好以下九个方面的工作：

　　（一）着力夯实万亿工业强市基础

　　明确工业发展目标。力争规模以上工业总产值突破7000亿元。其中汽车及零部件产业产值突破2100亿元，农产品加工业突破1700亿元，装备制造产业突破1100亿元，纺织服装产业突破630亿元，电子信息、医药化工产业分别突破530亿元和650亿元，节能环保、新能源新材料产业分别突破400亿元和260亿元；军民融合产业板块突破560亿元。力争全年新增1家产销过50亿元、10家过10亿元、20家过亿元的骨干企业，新增150家规模以上企业，新增中国驰名商标5件以上、湖北著名商标33件以上。高新区规模以上工业总产值达到2900亿元，襄州、枣阳分别突破900亿元，谷城突破800亿元，宜城、老河口分别突破700亿元。

　　加快推进工业项目建设。全年完成工业投资1650亿元，其中完成技改投资700亿元左右；实施亿元以上工业项目422个，力争建成投产180个以上。确保东风井关农机产业园一期、金鹰重工产业园二期、中车襄阳工业园、江山重工数字化核心制造基地、粤宜高科液晶显示器、华中医药产业园、鞋业产业园、正大百万头生猪产业化等37个投资过10亿元的重大项目竣工投产，确保际华生态纺织产业园、吉利农机装备产业园、高德红外应急安全装备产业基地、华宇汽车安全气囊等28个10亿元以上重大项目开工建设，确保三环精密锻造中心、新鄂西化工厂、长源东谷工业园、金洋冶金含铅固废物再生利用、德硕再生纸等21个10亿元以上在建项目顺利实施。围绕打造“中国新能源汽车之都”，突出抓好东风新能源汽车、鸿泰阳新能源汽车、骆驼股份动力电池等一批新能源汽车产业重大项目，力争新下线一款以上新能源轿车，新能源汽车产值实现翻番、突破120亿元。

　　强力推进工业招商。进一步推动招商重心向工业聚焦、招商力量向工业集中，深入实施产业链招商行动计划，重点突破汽车及新能源汽车、高端装备、现代农机装备、智能家电、农产品深加工、再生资源利用、日用轻工等产业链招商，力争全年招商引资实际投资额突破2000亿元，工业项目投资占比达到70%以上，力争新引进投资过5亿元的重大项目200个以上。

　　加大服务企业力度。牢固树立与企业一起“御寒过冬”的思想，全面落实加快推进万亿工业强市建设的十条政策措施，深入开展降低企业成本行动，坚持工业运行调度制度和现场办公制度，实行困难问题化解责任清单管理，扎实开展“千名干部进千企”活动，努力帮助企业排忧解难、减轻负担。

　　（二）实施加快发展现代服务业行动

　　突破性发展云服务业。加快建设华为大数据产业园，大力推进华为云服务华中大区中心、中国移动云计算中心二期、软通动力云应用等重点项目，集聚一批云服务和大数据产业链项目，加大云服务推广应用力度，着力打造面向全国、服务全省、辐射中部的“襄阳云谷”。

　　大力发展检测认证服务业。加快实施检测认证产业园二期工程，启动建设3个计量检测中心，确保5个省级检测中心投入运营、20家检测认证企业入驻园区，创建国家公共检验检测服务平台示范区和国家检验检测高技术服务业集聚区。

　　加快发展现代流通方式。开展“互联网+物流”行动，引导传统商贸物流企业利用新一代信息技术改造升级，建立线上线下融合发展新模式，加快推进自贸区跨境电商平台等8个重点项目建设，筹建进口商品展示交易中心直销店；加快建设市级农村商贸物流配送中心和乡镇商贸中心，推进电商进村、快递下乡。

　　加快发展文化旅游业。加快建设汉水文化旅游展示区等“一城两文化”标志性旅游项目，大力推进隆中创建5A级景区；着力培育乡村旅游、休闲度假游等旅游新业态，积极创建国家级自然保护区、森林公园、湿地公园，规划建设东津古镇、卧龙古镇等旅游小镇。

　　积极发展健康养老服务业。大力发展医养结合、社区养老、居家养老等养老服务模式，推动医院与养老机构开展合作，鼓励社会资本兴办养老机构，争创全省养老服务业综合改革试点。

　　（三）加快转变农业发展方式

　　提升综合生产能力。加大耕地保护力度，基本完成城市周边永久基本农田划定工作；加强农田水利建设，完成解决易旱地区干旱问题三年行动计划任务；大规模推进土地整理、中低产田改造和高标准农田建设，建成高产农田30万亩。开展“互联网+现代农业”行动，加快推进主要农作物生产全程机械化，大力推广应用农业新技术，大规模开展高产创建和绿色增产模式攻关，积极推行立体、循环、生态、高效种养模式，着力提高农产品生产质量和效益；加快建设中国有机谷“一核三区”，扩大有机农产品生产规模，新增“三品一标”农产品40个，确保核心区产业园展示中心、支持中心投入运营。

　　推进农业产业链建设。推动企业与农户结合、种养与加工结合、农业与旅游结合，大力培育“农户种养、就地加工、休闲旅游、循环生态”的一二三产业融合发展新业态，打造一批“种养—加工—旅游”一体化经营的产业综合体，大力支持谷城、南漳两个全国休闲农业和乡村旅游示范县建设。

　　创新农业经营方式。力争创建市级以上示范农民专业合作社100家、示范家庭农场100家、农村专业技术协会10家，培育新型职业农民5000人以上，促进农业适度规模经营，加快建立“管理在县、服务在乡”的农村公益性社会化服务新机制。扎实做好第三次全国农业普查工作。

　　（四）全力推进棚户区改造

　　坚定不移地把棚户区改造作为重大民生工程和发展工程，抢抓国家政策机遇，统一规划，分步实施，连片改造，强力攻坚。

　　加大棚户区改造投入力度。建立棚户区改造融资长效机制，加强与国开行、农发行等政策性金融机构的深度合作，充分发挥住房公积金的作用，探索政府与社会资本合作模式，拓宽棚户区改造融资渠道。力争全年完成棚户区改造投资350亿元，其中中心城区完成投资260亿元。

　　加快推进棚户区改造项目建设。全市新开工改造45227套，建成13092套；中心城区重点抓好襄城城南片区、樊城清河片区等11个改造项目，新开工改造19569套、建成4109套；推动棚户区改造向工矿区、城郊镇、重点镇覆盖。将棚户区改造配套基础设施建设纳入年度城建计划，与主体工程同步规划、同步报批、同步建设、同步交付使用，全面提升棚改片区建设品质和服务功能。建立棚户区改造项目审批快速通道，简化程序，限时审批，提高效率，确保项目快速落地开工。

　　创新棚户区改造征迁安置方式。坚持最大限度让利于民的原则，全面推行货币化安置，缩短安置周期，加快征迁进度，让群众尽快住上新房，满足群众多样化居住需求。对经济困难、无力购买新住房的棚户区居民，采取提供公共租赁住房的办法，满足其基本住房需求。将棚户区改造、保障性住房建设与化解房地产库存结合起来，通过政府购买库存商品房筹集保障房房源，扩大有效需求。

　　（五）强力推进重大基础设施建设

　　加快推进铁路建设。全力服务和推进汉十高铁、郑万高铁和蒙华铁路建设，全面完成征迁工作，加快建设东津高客站配套工程和蒙华铁路邓湖分解站，开工建设东津高铁枢纽和三座跨汉江铁路特大桥，全年完成铁路建设投资168亿元。

　　加快推进公路建设。开工建设保神高速和枣潜高速襄阳段，加快建设老谷高速跨汉江大桥，开工建设河谷、宜城两座跨汉江公路特大桥，全面完成316、207国道改造，新建改建一二级公路300公里、农村公路1000公里，全年完成公路建设投资140亿元。

　　加快推进机场和航运体系建设。确保襄阳机场航站楼10月份交付使用，飞行区及站前广场等配套工程年底投入使用。开工建设新集、雅口两座航电枢纽，加快推进襄阳新港小河港区、陈埠港区等重点项目建设。

　　加快推进重大能源和水利工程建设。扎实做好大唐热电联产、华电三期、路口电站等项目前期工作，大力推进4个风电、5个光伏发电、7个电网建设工程，全年完成能源投资50亿元。开工建设鄂北水资源配置16个分水口配套工程，继续实施大中型灌区节水配套改造。

　　（六）着力提升城市功能和宜居水平

　　加强城市新区建设。加快建设东津新区，规划建设高铁小镇；确保中心医院新院区、襄阳三中新校区等公共工程投入使用；加快庞公新区建设，基本建成“三纵五横一环”的骨干路网。

　　加强城市基础设施建设。重点抓好中心城区498个城建项目，力争完成投资550亿元。实施城市畅通工程，建成东外环及南北轴线，加快建设庞公大桥和苏岭山大桥，推进郑岗互通等11个绕城跨区通道工程，新建改造21条干支道路，新建改造4个公交枢纽站，新增公共停车泊位2500个以上；提升供水、供电、供气、排水、治污等市政设施服务功能，以“海绵城市”理念和方式推进城市建设，启动东津新区综合管廊建设试点。

　　加强山水园林城市建设。实施城市彩化试点工程，新建改建公园游园10个，新增城市绿地300万平方米、绿道30公里，全面推进市区滨江滨河道路综合整治，打造岸线近水亲水景观带。积极开展城市管理综合执法，完善城市数字化管理系统，建立第三方评价机制，扎实开展智慧城市建设试点，提高城市管理精细化水平。

　　加强生态环境建设。深入开展“蓝天碧水”行动，加快构建市、县、乡、村（社区）四级网格化、全覆盖的环境监管体系，全面完成国家“水十条”、“气十条”确定的年度目标任务，确保汉江襄阳段及饮用水源地水质持续稳定，全市空气质量明显改善；深入开展“绿满襄阳”行动，全面提升荒山、通道、村庄、社区、水系、引丹干渠等绿化水平；大力开展全民节能行动，推广应用新能源汽车300辆以上，新增绿色建筑20万平方米；加快发展以再生资源利用为主的循环经济，实施14个特色产业园区循环化改造；推进美丽宜居乡村建设，打造一批国家级和省级试点示范。

　　（七）推动大众创业万众创新蓬勃发展

　　优化“双创”环境。推进工商登记便利化，探索集群注册、一址多照、同城通办等新型登记模式，推动企业登记管理全程电子化。全面落实财税金融扶持政策，设立创新创业引导基金、天使基金，开展小额股权众筹试点，大力引进和培育风投、创投等投资机构，建立“双创”融资担保机制。加快建设市县两级“双创”服务中心，打造服务超市，完善服务功能。

　　搭建“双创”平台。运用“互联网+”打造线上线下相结合的“双创”新载体，依托科技孵化器、创业基地、大学生创业园、院士专家创新创业园等建设专业众创空间，加快建设襄阳创业大街、东津新区创业街，实现县（市、区）、开发区专业众创空间全覆盖。鼓励互联网企业搭建网络众创平台，支持骨干企业搭建员工众创平台，构建开放式创业创新体系。

　　培育“双创”主体。聘请国内外成功创业者、知名企业家、天使和创业投资人、专家学者组建导师团队，开展高水平、专业化的“双创”辅导。实施大学生创业引领计划、科研人员创业支持计划、高端创业创新人才引进计划、返乡创业促进计划，力争全年引进高端人才创业创新团队8个，新增科技型小微企业300家以上、大学生创业者2000人以上，新发展“双创”市场主体8万户以上。

　　强化科技创新。深入推进国家创新型试点城市建设，着力打造科技创新平台，力争在引进新的产业研究院（中心）上取得实质性突破，确保华科工研院投入运营，新增省级以上研发创新平台10个。大力推进协同创新，争创一批国家级和省级产业技术创新联盟，扎实抓好70个产学研合作项目。大力推进科技成果转化，扎实抓好国家技术转移中部中心襄阳分中心建设，完成技术合同交易额85亿元。大力支持企业自主创新，争取实施省级以上科技计划项目100个，完成省级科技成果登记190项，新认定高新技术企业30家以上。实施更加积极的人才政策，完善“隆中人才支持计划”，以人才创新促进科技创新。

　　（八）持续深化改革扩大开放

　　深化行政管理体制改革。持续简政放权，完善“三个一”管理体制，健全“一局两委”工作机制，完善权力清单、责任清单、市场协同监管清单管理制度，建立政务服务事项清单、中介服务事项清单制度。推进各审批系统无缝对接、市县乡村政务服务平台互通共享，推动政务服务向基层延伸。完成市直重点行业领域综合执法体制改革，深化“区镇合一”体制改革，推进事业单位行政职能回归机关，推动生产经营类事业单位转企改制。

　　深化投融资体制改革。做大汉江产业基金和城市发展基金规模，撬动金融资本、社会资本投入实体经济和城市建设。推动政府投融资平台加快转型发展，构建集“融资、投资、建设、偿债”功能为一体的投融资主体，全年力争获批国家专项债券70亿元。创新公共服务项目和基础设施投融资机制，重点推进12个PPP项目。加快发展多层次资本市场，力争新增1家上市企业、15家新三板挂牌企业、50家四板挂牌企业。完善政银企合作机制，提升市中小企业担保公司、农业产业化担保公司担保能力，鼓励银行业金融机构增加信贷规模、创新金融产品和服务，力争新增贷款余额260亿元。

　　深化城镇化发展体制机制改革。全面落实“四零门槛”为核心的户改新政，以居住证为载体建立基本公共服务机制，初步实现基本公共服务城镇常住人口全覆盖；扎实办好宜城全国新型城镇化综合改革试点，打造全国农村宅基地制度改革的“湖北样本”；全面完成不动产登记制度改革和农村土地确权登记颁证。

　　深化财政和国有资产管理体制改革。实行中长期财政规划管理，抓好编制三年滚动财政规划试点工作；规范公共资源交易管理，整合建立统一的公共资源交易平台。以管资本为主加强国有资产监管，推进经营性国有资产集中统一监管，支持国有企业发展混合所有制经济。

　　深入推进开放开发。扎实做好湖北自贸区襄阳片区筹建工作，加快推进保税物流中心建设，建成综合服务中心、海关监管仓库、保税物流仓库及信息系统，确保封关运行；启动建设铁路物流中心和公路港，积极申报一类航空口岸。打通连接亚太的“铁水联运”、“公水联运”通道，开通连接欧亚大陆桥的“铁铁联运”通道。积极开展国际产业合作，支持优势企业在境外开展并购重组、股权投资、创业投资；全面实行准入前国民待遇加负面清单管理制度，优化营商环境，吸引外商投资。着力提高对外贸易水平，加强国家和省级出口基地（示范区）建设，积极发展加工贸易。

　　（九）努力提高民生保障水平

　　努力为人民群众提供更多更稳定的就业增收机会。全面落实更加积极的就业政策，提供优质高效就业服务，加强职业技能培训，提高劳动者就业择业创业能力，千方百计促进各类群体就业，全年城镇新增就业8.2万人，创业带动就业1.3万人。

　　努力让贫困群众分享更多的发展成果。扎实推进脱贫攻坚三年行动计划，全面落实脱贫工作责任制，深入开展脱贫“五帮”活动，大力实施“五个一批”脱贫攻坚工程，加快推进“六到农家”基础设施改善工程，确保全年实现140个贫困村出列、减贫11万人。

　　努力办好更高水平的人民满意教育。着力提升基础教育办学能力，新建、改扩建公办幼儿园35所，继续推进农村寄宿制学校“五有配套”和义务教育学校标准化建设，深入推进义务教育学校校长、教师轮岗交流，加快实施优质高中增量工程，扎实做好省级教育信息化试点工作。优化职业教育布局，组建职教联盟。支持湖北文理学院提高办学水平，支持襄阳职业技术学院扩大四年制高等职业教育试点专业，确保襄阳汽车职业技术学院办学条件通过省级“合格评估”。

　　努力让人民群众享有更高水平的公共文化服务。加快构建覆盖城乡的公共文化体育服务网络，加强青少年综合服务平台和群众文化活动阵地建设，确保成功创建国家公共文化服务体系示范区。持续推进文化体育服务下乡，广泛开展“书香溢襄阳”全民阅读和全民健身活动，着力打造品牌文化体育赛事。加大历史文化遗产保护力度，扎实做好古城墙和万里茶道节点申遗工作。

　　努力让人民群众享有更高质量的医疗卫生服务。持续深化县级公立医院综合改革，健全完善分级诊疗制度，全面推进医疗联合体建设，深入实施医疗机构服务能力提升工程；切实加强公共卫生服务能力建设，建立全市人口健康信息平台，积极推行居民健康卡。推动计划生育工作转型发展和服务管理改革。

　　努力让人民群众享有更加公平的社会保障。全面实施“全民参保登记计划”，推进职工和城乡居民全面持续参保。加大社会救助力度，完善社会救助标准与人均消费支出相适应的动态调整机制，认真落实困难残疾人生活补贴和重度残疾人护理补贴制度，加强农村福利院建设，全面开展重特大疾病医疗救助。加强养老服务体系建设，确保城市社区居家养老服务中心实现全覆盖，农村社区老年互助照料中心覆盖率达到60%。

　　努力为人民群众营造更加安全和谐的社会环境。编制实施“七五”普法规划，深化公安和司法行政改革，加快建设立体化社会治安防控体系，健全完善社会矛盾大调解体系，推进行政复议委员会规范运行，提升城乡社区网格化管理服务能力，着力打造全国平安建设示范区，努力争创全国综治“长安杯”，积极创建全国法治城市。全面落实安全生产责任制，扎实开展隐患排查治理标准化、信息化体系建设，坚决遏制重特大事故发生，积极创建全国安全发展示范城市。健全食品药品安全治理体系，大力实施食品放心工程，积极创建国家食品安全城市。认真做好国防动员工作，大力支持军队改革和国防后备力量建设，积极创建国家和省“双拥”模范城。加强民族宗教工作，扎实做好对口支援工作。

　　新的一年，我们将切实加强政府自身建设。坚持科学理政。加快转变政府职能，深入推进政府工作理念创新、服务方式创新，提升政府治理能力和水平，打造高效和服务型政府。坚持依法行政。自觉运用法治思维和法治方式履行政府职能，扎实抓好依法行政示范区创建，办好全省法治政府建设试点，认真做好政府立法工作；自觉接受市人大及其常委会的法律监督、工作监督和市政协的民主监督，加强行政监察、审计监督和政务公开，让权力在阳光下运行。坚持从严治政。认真落实“三严三实”要求，弛而不息整治“四风”，重拳整治为官不为、懒政怠政，牢固树立雷厉风行、主动作为、狠抓落实的优良政风；把纪律和规矩挺在前面，严格落实中央八项规定精神，按照只减不增的要求从严控制“三公经费”预算和财政供养人员编制；压实主体责任，落实“一岗双责”，深入推进政府系统党风廉政建设，着力打造清廉政府。

　　各位代表，我们肩负着加快建设汉江流域中心城市的历史重任，承载着全市人民对美好生活的殷切期待。让我们在市委的坚强领导下，砥砺奋进，跨越前行，共同谱写“十三五”发展的新华章，为率先全面建成小康社会而奋斗！
